# Spatial Analysis of COVID-19 Vaccination: A Scoping Review

**DOI:** 10.3390/ijerph182212024

**Published:** 2021-11-16

**Authors:** Abolfazl Mollalo, Alireza Mohammadi, Sara Mavaddati, Behzad Kiani

**Affiliations:** 1 Department of Public Health and Prevention Science, School of Health Sciences, Baldwin Wallace University, Berea, OH 44017, USA; amollalo@bw.edu; 2 Department of Geography and Urban Planning, Faculty of Social Sciences, University of Mohaghegh Ardabili, Ardabil 56199, Iran; a.mohammadi@uma.ac.ir; 3 Faculty of Medicine & Surgery, Policlinic University Hospital of Bari Aldo Moro, 70124 Bari, Italy; s.mavaddati@studenti.uniba.it; 4 Department of Medical Informatics, School of Medicine, Mashhad University of Medical Sciences, Mashhad 91779, Iran

**Keywords:** COVID-19, GIS, spatial analysis, scoping review, vaccination

## Abstract

Spatial analysis of COVID-19 vaccination research is increasing in recent literature due to the availability of COVID-19 vaccination data that usually contain location components. However, to our knowledge, no previous study has provided a comprehensive review of this research area. Therefore, in this scoping review, we examined the breadth of spatial and spatiotemporal vaccination studies to summarize previous findings, highlight research gaps, and provide guidelines for future research. We performed this review according to the five-stage methodological framework developed by Arksey and O’Malley. We screened all articles published in PubMed/MEDLINE, Scopus, and Web of Science databases, as of 21 September 2021, that had employed at least one form of spatial analysis of COVID-19 vaccination. In total, 36 articles met the inclusion criteria and were organized into four main themes: disease surveillance (n = 35); risk analysis (n = 14); health access (n = 16); and community health profiling (n = 2). Our findings suggested that most studies utilized preliminary spatial analysis techniques, such as disease mapping, which might not lead to robust inferences. Moreover, few studies addressed data quality, modifiable areal unit problems, and spatial dependence, highlighting the need for more sophisticated spatial and spatiotemporal analysis techniques.

## 1. Introduction

Coronavirus disease-2019 (COVID-19), caused by severe acute respiratory syndrome coronavirus 2 (SARS-CoV-2), has been a substantial threat to global public health [1,2]. On 11 March 2020, the disease was declared a pandemic by the World Health Organization (WHO) [3]. Despite worldwide endeavors to control the pandemic, the morbidity and mortality rates of the disease have not significantly declined [4]. Moreover, the outbreak adversely impacted the global economy, which may take several years to recover [5]. As of 5 October 2021, over 236 million incident cases and 4.83 million deaths have been reported worldwide [6]. In addition, recent estimations project that the ongoing global outbreak is far from over [7].

Recent evidence suggests that fully vaccinated individuals get better protection against the virus [8]. According to the Center for Disease Control and Prevention (CDC), unvaccinated people are more than 5 and 29 times as likely as fully vaccinated people to get the infection and be hospitalized due to the virus, respectively [9]. The first experimental COVID-19 vaccine doses were administered to humans in March 2020, and continued with a global race to curb the spread of the virus by differing levels of vaccine rollout [10]. Achieving global vaccine coverage remains a significant equity hurdle and is an essential part of controlling the outbreak [11]. Lack of access to vaccines in most low- and middle-income countries is one of the main reasons for low vaccination rates. Vaccine hesitancy in most high-income countries, mainly due to safety concerns in vaccine development, lack of confidence in vaccine efficacy, potential side effects of vaccination, the spread of misinformation, and mistrust of the government and public health system, has contributed to low vaccination rates [12,13].

Spatial and spatiotemporal analyses are practical approaches to support public health decision-makers in monitoring COVID-19 vaccination coverage over time for targeted interventions and strategic action plans [14]. The techniques can identify the areas and populations at the highest priority for immunization by vaccines, particularly in association with determinant socioeconomic factors [15]. Although spatial and spatiotemporal analyses have been commonly performed in the study of morbidity [16], infection [17], and mortality [18,19] of COVD-19, fewer studies have examined spatial heterogeneity of COVID-19 vaccination.

Although a growing body of literature have utilized spatial and spatiotemporal approaches for COVID-19 vaccination research in different parts of the world, to our knowledge, no previous study has synthesized the geospatial studies concerning COVID-19 vaccination. To address this research gap, we aimed to conduct a comprehensive scoping review on the application of spatial analysis in COVID-19 vaccination research. In the absence of some of the more recent SARS-CoV-2 variants in earlier studies, reflecting the spatial analysis of COVID-19 vaccination based on the timely findings is necessary.

## 2. Materials and Methods

This review was undertaken according to a five-stage methodological framework developed by Arksey and O’Malley [20]. The following is a summary of each stage.

### 2.1. Stage 1: Identifying the Research Question

The primary objective of this research was to identify the extent of previous spatial and spatiotemporal analyses that contributed to COVID-19 vaccination research. Moreover, we aimed to synthesize the findings, identify the research gaps and limitations, and provide guidelines for future research.

### 2.2. Stage 2: Identifying Studies

We systematically searched all published articles in PubMed/MEDLINE, Scopus, and Web of Science search engines. For additional studies beyond the scope of the databases, reference searching was manually carried out. We searched articles from inception to 21 September 2021, with no geographic limitations. Table 1 summarizes the list of keywords and medical subject headings (MeSH) terms to capture all COVID-19 vaccination studies that applied any spatial or spatiotemporal analysis/technique. The Boolean operators (AND, OR) were used to synthesize search terms. The retrieved articles based on the keywords or MeSH terms were uploaded into Endnote X9 reference manager software (Clarivate Analytics, Philadelphia, PA, USA), where duplicate records were removed for further screening. Table 1 provides the search strategy used in the PubMed database. Detailed information regarding search strategies in Scopus and Web of Science databases is presented in Supplementary File S1.

### 2.3. Stage 3: Selecting Studies

The articles for this review were eligible for inclusion if they met the following criteria: (a) were written in English; (b) were peer-reviewed original journal articles; (c) were focused on any aspect of COVID-19 vaccination; (d) were involved in at least one spatial or spatio-temporal analysis tool or technique. However, we excluded non-peer-reviewed articles (e.g., letters, editorials, reviews, commentaries, grey literature), the studies that did not focus on COVID-19 vaccination, and studies that did not involve any spatial analysis/technique.

Based on the title and abstract screening, the eligibility of each article against the above-stated criteria was evaluated by two reviewers (A.M. and B.K.) independently. After excluding irrelevant articles, full texts of all the remaining records were scrutinized to ensure the primarily selected papers had addressed research objectives. A third reviewer was consulted to resolve possible conflicts, and upon discussion, a consensus on inclusion or exclusion was made.

### 2.4. Stage 4: Charting the Data

After the articles were selected for inclusion in the final analysis, two excel spreadsheets were used to chart the data independently by two reviewers (A.M. and B.K.). For each article chosen, the following items were extracted: title; year; location; sample/populations size; the scale of analysis (e.g., city, state, country); research method (exploratory, descriptive, explanatory, modeling); geospatial level of complexity; spatial analysis method; visualization software; variables; key findings; and data sources. Using these standard forms, the reviewers continuously charted the data independently. Any disagreement was reconciled by discussion.

According to an article by Nykiforuk and Flaman [21], the selected articles were classified into the following themes: (1) disease surveillance (i.e., vaccine mapping and vaccine modeling); (2) risk analysis; (3) health access and planning (i.e., access to vaccine services and vaccine resource management); and (4) community health profiling. Moreover, according to the Grekousis [22] article, the studies’ geospatial and visualization levels of complexity were scored from zero to seven. The scores were based on the presence of the following criteria: without any complexity (score = 0); descriptive (score = 1); exploratory (score = 2); descriptive and exploratory (score = 3); explanatory and modeling (score = 4); descriptive, explanatory, and modeling (score = 5); exploratory, explanatory, and modeling (score = 6); descriptive, exploratory, explanatory, and modeling (score = 7).

### 2.5. Stage 5: Collating, Summarizing, and Reporting Results

The distribution of studies was mapped in ArcGIS Desktop software (ESRI, Redlands, CA, USA). The appropriate graphs/charts were generated in Microsoft Excel to summarize the status of vaccination research from a geospatial perspective, and to highlight research gaps for future studies.

The detailed structure of our review article has been explained in Supplementary File S2 according to the Preferred Reporting Items for Systematic reviews and Meta-Analyses extension for Scoping Reviews (PRISMA-ScR) Checklist.

## 3. Results

### 3.1. Study Characteristics

**Figure 1:**
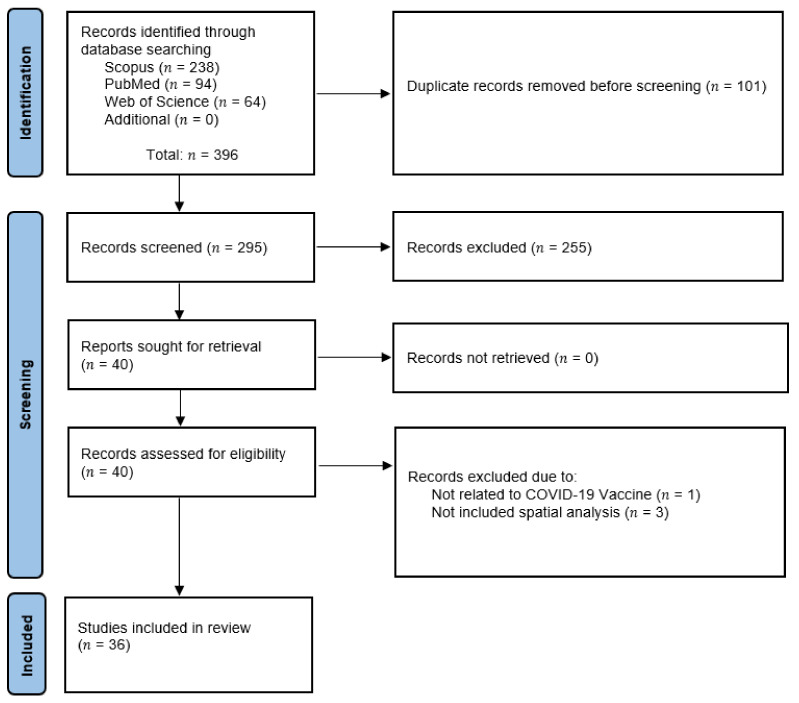
Study selection flow diagram.

During the initial literature search from inception to 21 September 2021, we identified 396 articles in PubMed (n = 94), Scopus (n = 238), and Web of Science (n = 64) databases. However, no additional unique articles were found from reference searching. After exclusion of duplicates (n = 101) and irrelevant studies (n = 255), the full texts of remained articles (n = 40) were assessed for inclusion eligibility. Among the 40 remaining studies, we excluded four articles: three articles which did not involve any spatial analysis; and one article that was unrelated to the COVID-19 vaccine. Therefore, in total, 36 articles met the inclusion criteria for our review. Figure 1 depicts the Preferred Reporting Items for Systematic Reviews and Meta-Analyses (PRISMA) flow diagram summarizing the article selection process.

**Figure 2:**
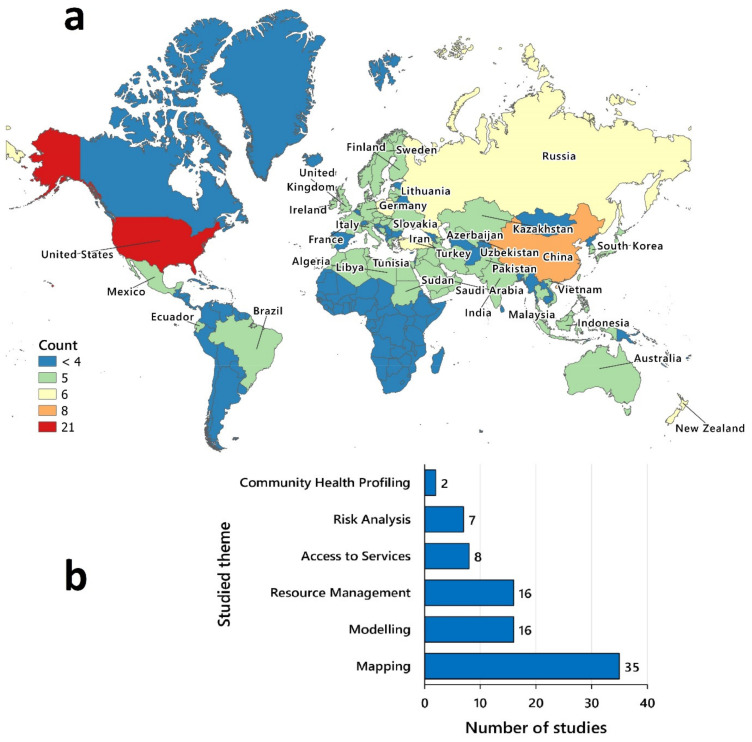
The number of selected studies based on (**a**) geographic distribution and (**b**) spatial analysis theme.

The selected articles were published in 2021 (n = 33, 91.67%), and 2020 (n = 3, 8.33%). The geographic distribution of articles was mostly concentrated in the US (n = 21, 58.33%), China (n = 9, 25.00%), and New Zealand (n = 8, 22.22%). Figure 2a shows the number of studies for the locations that they were carried out. The geospatial and visualization level of complexity of the studies were scored from 1 to 7 (median = 3, standard deviation = 1.26). Most articles (n = 26, 72.22%) had a level of complexity of 3 or less: six articles (score = 1); one article (score = 2); and nineteen articles (score = 3). Fewer articles (n = 10, 27.78%) had a score above 3: seven articles (score = 4); two articles (score = 5); and one article (score = 7). Table 2 summarizes the geospatial and visualization level of complexity for each article.

There were only n = 6 (16.67%) studies that used a single research method: one exploratory (2.78%); and five descriptive (13.89%) studies. Most articles (n = 26, 72.22%) used a combination of two research methods: a hybrid of descriptive and exploratory (n = 19, 52.78%); and a mixture of explanatory and modeling (n = 7, 19.44%). Among articles that used a combination of three research methods, one article (n = 1, 2.78%) used a hybrid of descriptive, explanatory, and exploratory methods, and two articles (n = 2, 5.56%) used a mixture of descriptive, explanatory, and modeling methodologies. Only one article (n = 1, 2.78%) employed all four research methods. Table 2 presents the methodological approach used for each article.

It should be emphasized that although the articles were categorized into separate spatial analysis themes (i.e., disease surveillance, risk analysis, health access and planning, and community health profiling), they were not mutually exclusive. For example, many articles that included disease surveillance also analyzed health access and planning. In this review, when more than one theme was discussed in a study, the article was assigned to the most closely related themes. Figure 2b shows the number of articles in each theme. The following is a summary of the findings of the most related articles for each category. Furthermore, a summary of key findings related to each category has been provided in Supplementary File S3.

### 3.2. Disease Surveillance

#### 3.2.1. Vaccine Mapping

Almost all articles (n = 35, 97.22%) used at least one type of thematic mapping of COVID-19 vaccination. The articles mainly represented geospatial distribution of vaccination, and the output of predictive analyses or models. The employed maps were usually heat maps, choropleth maps, or inset maps. However, the Voronoi map (Theisen polygon) was utilized in only one study [23]. The contribution of 11 articles to the spatial analysis of COVID-19 vaccination was only limited to mapping [24,25,26,27,28,29,30,31,32,33,34]. Although most mapping studies disregarded time components, and used static presentation of data, temporal variations or transmission dynamics were mapped in [24,29,30,35,36,37].

On a global scale, Liu and Liu [30] collected a relatively large sample (n = 2,678,372) of data from Twitter. Using heat maps, they concluded that public sentiment on COVID-19 vaccines varied significantly over time and space. Similarly, Guntuku et al. [35] utilized choropleth maps to describe geographical and temporal variations of COVID-19 vaccine concerns in the US based on vaccine-related tweets. Their findings suggested that Twitter discourse regarding COVID-19 vaccines in the US varies significantly across different communities, and changes over time.

In a web-based study in the US, Mast et al. [36] developed novel web-based methods to communicate location and time-based vaccine uptake to monitor the impact of existing and new vaccines. The maps could identify geographic hotspots with low vaccination rates and persistent disease rates over time. A census-tract level study in Texas [38] indicated that real-time geospatial analysis is necessary to identify vaccination gaps, and to rapidly increase vaccination uptake to reach herd immunity in the vulnerable and vaccine-hesitant groups.

In a spatiotemporal study, Ali et al. [24] examined public perceptions of COVID-19 vaccines using choropleth maps. They found that fear sentiment remained unchanged in populous US states, whereas trust sentiment declined slightly in the same states. Moreover, changes in emotions were most notable among less populated states in the central US. Similarly, in this country, Hu et al. [29] used choropleth maps, and observed an increasing trend in positive sentiments and a decrease in negative public opinions towards vaccines in most states, reflecting the rising confidence and anticipation of the public towards vaccines.

#### 3.2.2. Vaccine Modeling

Disease modeling expands disease mapping when mapping is combined with spatial statistical analysis. The main purposes of disease modeling are to predict or forecast disease status, and to identify significant factors, high-risk areas, or gaps to generate policies for targeted immunizations [21]. The articles in this category utilized geostatistical and modeling techniques, such as agent-based models based on individual, socioeconomic, and environmental variables.

In an earlier study in 2020, Alouane et al. [39] examined hotspot mutations in 30,983 SARS-CoV-2 genomes to deepen the understanding of the intra-genomic diversity of SARS-CoV-2. They found that, unlike the influenza virus or HIV viruses, SARS-CoV-2 has a low mutation rate, making developing an effective global vaccine very likely.

In China, Zhang et al. [40] used an agent-based susceptible-exposed-infectious-removed (SEIR) model to analyze the efficiency of various intervention strategies in preventing infection caused by the SARS-CoV-2 virus. They suggested that to reduce transmission, students should be prioritized for vaccination rather than retired older people and preschool-aged children. They suggested that home isolation and taking the nucleic acid test immediately after symptom onset were more effective than wearing masks in public places.

A study in the US [41] used SaTScan software to optimize equitable distribution of the COVID-19 vaccine. The study suggested that a targeted geospatial approach is necessary to maximize vaccine rollout efficiency by including high-risk populations that may otherwise be subjected to delays or missed vaccination. In another study in the US, Shastri et al. [42] measured the impact of the COVID-19 pandemic, and obstetrician and gynecologist workforce distribution on vaccine deployment. They indicated that during the planning and executing vaccine allocation, especially in the early stages of distribution, it is critical to evaluate which communities can benefit from the limited number of vaccines. They found that older physicians practicing in North Dakota, South Dakota, and Iowa experience the highest burden of COVID-19 caseloads compared to all other states.

### 3.3. Risk Analysis

The articles that analyzed the risk of vaccination (assessment, management, communication, or monitoring) mainly examined the risk regarding demographic and socioeconomic factors, including age, race, occupation, and income level. Moreover, the distribution and allocation of vaccines for high-risk groups was analyzed. The geographic distribution of all articles in this category was restricted to the US.

In a state-level study in the US, Al Rifai et al. [43] assessed socioeconomic determinants of influenza vaccination by race/ethnicity. They found that Blacks and Hispanics have been disproportionately affected by COVID-19. Moreover, receiving an influenza vaccine was of particular importance to mitigate the risk associated with overlapping influenza and COVID-19 infections.

The spatiotemporal trends in COVID-19 death rates in the US was examined by [37]. They also investigated the effects of COVID-19 time-dependent and social vulnerability factors on COVID-19 death rates. Their findings demonstrated that over the three phases of the pandemic (first wave, second wave, and post-vaccine deployment), hot spots have shifted from densely populated cities and the states with a high percentage of socially vulnerable individuals to the states with relatively relaxed social distancing requirements, and then to the states with low vaccination rates. Similarly, Kandula and Shaman [44] investigated the associations between COVID-19 mortality and population-level health and socioeconomic indicators. They concluded that a significant spatial autocorrelation exists in COVID-19 mortality, and population health/socioeconomic indicators account for considerable variability in county-level mortality. They suggested that national and subnational socioeconomic indicators and burden of disease estimates can potentially be leveraged to allocate vaccines optimally to reduce severe outcomes.

Using a multiscale geographically weighted regression model, Mollalo and Tatar [45] analyzed spatial heterogeneity of COVID-19 full-vaccination rates across all counties in the continental US using social vulnerability index data. They found that the model explains over 79% of the variance of vaccination rate based on per capita income and minority (%) (with positive impacts), and age 17 and younger (%), mobile homes (%), and uninsured people (%) (with negative effects).

Using regularly available demographic information, Malik et al. [46] predicted COVID-19 vaccine acceptance, and identified the most vulnerable populations for public health officials and politicians to develop messaging for all Americans while targeting communities most in need. They found that there are noticeable demographic and geographical disparities in vaccine acceptance.

### 3.4. Health Access and Planning

Health access and planning was the second most studied theme regarding spatial analysis of COVID-19 vaccination. The articles assessed the accessibility of different social groups to the healthcare facilities that provide vaccination. In total, 16 articles (44.4%) examined this type of analysis in various parts of the world at the regional, state, and national levels.

#### 3.4.1. Access to Vaccine Services

In Brazil, Rocha et al. [47] explored how spatial artificial intelligence can help health managers to effectively implement the national COVID-19 vaccination plan. They found that around 18% of Brazil’s elderly population lived more than 4 km from a vaccination point. Moreover, the number of primary care centers located more than 5 km from base transceiver stations that provided internet access was largest in the north and northeast regions.

Through the introduction of community-based walk-up sites in New Orleans, US, Hernandez et al. [48] evaluated the increase in vaccination coverage and the use of COVID-19 testing services for vulnerable and hard-to-reach populations. They concluded that, even though inequalities appeared at the metropolitan scale, mobile and walk-up sites improved both coverage and accessibility to COVID-19 testing services for hard-to-reach populations.

A study in Aotearoa, New Zealand [49] examined the equity implications of the geographic distribution of COVID-19 vaccine delivery locations. They found that several areas with significant travel times to potential vaccine delivery sites had an elevated risk of COVID-19 disease and severity. Similarly, from the same study area, Whitehead et al. [50] examined the spatial and associated health equity implications of the geographic distribution of COVID-19 vaccination services. They noted that spatial accessibility to vaccination services varied across Aotearoa, and appeared better in major cities than in rural regions.

In the second-most populous city in Iran, Mohammadi et al. [51] measured potential spatial access to COVID-19 vaccination centers. They found that the periphery and poor areas of the city had the least access to COVID-19 vaccination centers. They suggested that equitable COVID-19 vaccination services in metropolitan areas should also include transportation networks and spatial access in modeling.

#### 3.4.2. Vaccine Resource Management

Although the articles that addressed access to vaccine services also examined vaccine resource management, several studies explicitly addressed vaccine resource management.

On a global scale, Wang et al. [52] aimed at providing global, regional, and national estimates of target population sizes for vaccine allocation to inform country-specific immunization strategies. This study presented a strategy for vaccine allocation based on three main goals, which should serve as a general framework when discussing and evaluating plans.

Cot et al. [27] emphasized the use of neighborhood-scale geospatial tools and data to address the challenge of inequality in vaccine distribution. They implied that neighborhood-level geospatial data would continue to aid public health in striving toward a greater and more equitable health system. In this regard, Krzysztofowicz and Osińska-Skotak [23] showed that geographic information system (GIS) technology allows practitioners to quickly analyze various vaccination scenarios and optimize the delivery of vaccine doses. Moreover, they examined the possibility of using GIS technology in Poland to quickly analyze various vaccination scenarios and optimize the delivery of vaccine doses. They indicated that this technology allowed practitioners to design efficient distribution scenarios rapidly. In Australia, Shahparvari et al. [53] used a geo-targeted approach to achieve vaccination of the entire population with less logistical resources. The approach enhanced spatial accessibility to vaccine centers by creating priority-based zoning.

One of the main criteria for equitable vaccine distribution is predicting the geographic distribution of the active virus at the time of vaccination. In the US, Davahli et al. [54] employed a self-organizing map, long short-term memory (LSTM) model, recurrent neural network model, and stochastic mixture density network model to predict the behavior of the COVID-19 pandemic. They concluded that the deterministic LSTM model could predict the geographic spread of active virus with considerable accuracy.

Using mobile phone trajectory data, Zhou et al. [55] used an integrated spatial model of an agent-based model and SEIR to examine the spatial heterogeneity of COVID-19 transmission in China. They also optimized vaccine distribution strategies considering spatial prioritization. Their results demonstrated the importance of space in optimizing vaccines allocation, and highlighted that space-and-age strategy greatly improves the effectiveness of vaccine usability.

### 3.5. Community Health Profiling

There were only two studies (5.56%) that examined community health profiling of COVID-19 vaccination. In this category, the relationship of COVID-19 prevalence with other vaccination coverages, such as Bacillus Calmette–Guérin (BCG) and influenza, were assessed.

In Ecuador, Garzon-Chavez et al. [56] used Mann–Kendall statistics to identify a possible link between BCG vaccination coverage and COVID-19. They found that the BCG vaccination coverage was low in 50% of Ecuadorians, and a high prevalence of COVID-19 was identified in the geographic areas with low coverage of BCG vaccination—thus, low-coverage areas were more affected by COVID-19.

Using Google and several websites of official public health agencies across 51 nations, [57] aimed to extend a previous ecological study of the effects of vaccines on COVID-19 cases and death rates. They also tested possible additive and synergistic vaccination effects of COVID-19 prophylaxis for controlling co-variance among vaccines. They found significant positive correlations between invasive pneumococcal disease and COVID-19 rates, whereas significant negative correlations were found between pneumococcal vaccination and COVID-19 rates.

**Table 2:** Characteristics of the included studies.

Study	Disease Surveillance		Risk Analysis	Access and Resource Management		Community Profiling	Research Method *	Geospatial Complexity Score
	Disease Mapping	Disease Modelling		Access to Healthcare	Resource Management			
Liu and Liu [30]	✓						R	2
Zhang, Chan [40]	✓	✓					N, M	4
Mast, Heyman [36]	✓	✓					N, M	4
Al Rifai, Khalid [43]	✓		✓				D, R	3
Solomon, Hsieh [41]	✓	✓					D, R	1
Nguyen, Nguyen [32]	✓						D, R	3
Wang, Wu [52]	✓				✓		D	1
Shastri, Rasendran [42]	✓				✓		D	1
Rocha, Boitrago [47]	✓	✓		✓	✓		D, R	3
Park, Kearney [37]	✓	✓	✓		✓		D, N, R	3
Rodríguez-Vidales, Garza-Carrillo [34]	✓						D	1
Kandula and Shaman [44]	✓	✓	✓		✓		D, R	3
Davahli, Karwowski [54]	✓				✓		N, M	4
Malik, McFadden [46]	✓		✓		✓		N, M	4
Guntuku, Buttenheim [35]	✓		✓				D, R	3
Chakraborty, Sharma [25]	✓						D, R	3
Alouane, Laamarti [39]	✓	✓					D, R	3
Hernandez, Karletsos [48]	✓		✓	✓	✓		D, R	3
Cot, Cacciapaglia [27]	✓						D, R	3
Garzon-Chavez, Rivas-Condo [56]	✓					✓	D, R	3
Mohammadi, Mollalo [51]	✓	✓		✓	✓		N, M	4
Shahparvari, Hasanizadeh [53]	✓	✓		✓	✓		N, M	4
Root-Bernstein [57]	✓					✓	D, R	3
Whitehead, Scott [49]	✓	✓		✓	✓		D, R	3
Whitehead, Carr [50]	✓	✓		✓	✓		D, R	3
Bauer, Zhang [38]	✓	✓			✓		D, R	3
Chakraborty, Sharma [26]	✓						D, R	3
Qunaibi, Basheti [33]	✓						D, R	3
Lei [58]	✓			✓	✓		D	1
Zhou, Zhou [55]	✓	✓			✓		D, R, N, M	7
Ali, Rahman [24]	✓						D, R	3
Hu, Wang [29]	✓						D	1
Grauer, Löwen [28]		✓					N, M	4
Krzysztofowicz and Osińska-Skotak [23]	✓	✓		✓	✓		D, N, M	5
Marcec, Majta [31]	✓						D, R	3
Mollalo and Tatar [45]	✓	✓	✓				D, N, M	5

✓ indicates the theme used in the study. * D—Descriptive; R—Exploratory; N—Explanatory; M—Modeling.

## 4. Discussion

To our knowledge, this scoping review is the first study that synthesized the applications of spatial and spatiotemporal analyses and tools in the study of COVID-19 vaccination. This timely study screened all peer-reviewed articles (published before 21 September 2021, in PubMed/MEDLINE, Scopus, and Web of Science search engines) that utilized at least one type of spatial analysis tool or technique. We found 36 articles that met the inclusion criteria, primarily concentrated in the US, whereas very few specific studies were reported from African and South American countries. The fewer studies mainly in developing and underdeveloped countries might be due to significantly lower vaccination rates than in high-income countries [59]. Additionally, the number of studies with geographic locations outside the US was insufficient for spatial epidemiological inferences. Although the spatial analysis themes were not mutually exclusive, this review could identify research gaps for future spatial analysis of COVID-19 vaccination.

Unlike many spatial and spatiotemporal studies that have analyzed COVID-19 morbidities and mortalities using sophisticated techniques, our findings suggested that most COVID-19 vaccination studies utilized rudimentary spatial operations centered on disease surveillance themes. However, there were infrequent studies regarding health profiling, most likely because it’s a newer area of research in public health [21]. As various types of COVID-19 vaccines are becoming more available worldwide, especially in low and middle-income countries, the location-based vaccination data will also be more available, and a growing number of literatures investigating the vaccination distribution from a spatial perspective is expected. Although our findings indicated that spatial accessibility to vaccination centers and resources is becoming more prevalent in the recent literature, a higher level of spatial analysis to enhance policy makers insights of vaccination programs is required.

Almost all selected studies relied on some form of thematic mapping primarily based on choropleth and heat maps, probably because vaccination data are usually aggregated at the regional, state, and national levels due to privacy reasons. Although these maps can visualize the spatial pattern of vaccination, and are helpful in identifying high/low vaccinated areas for further planning, they are susceptible to mislead decision-makers due to modifiable areal unit problems (MAUP) [60]. Although previous studies have discussed why MAUP can bias findings, and how to alleviate its effect (for example, by using Bayesian modeling [61,62]), none of the selected studies in this review considered this an important source of error. MAUP can also occur in space-time analysis when the data are collected differently over time, and for socioeconomic variables that are often presented at the aggregated levels, particularly in accessibility and resource management studies. Additionally, most selected studies did not evaluate or involve spatial autocorrelation in their analysis, leading to erroneous conclusions. In most studies, we observed a lack of analyzing MAUP and spatial autocorrelation, highlighting the need to validate findings with independent datasets.

Several studies used Twitter as the source of data for their analysis [24,29,30,35]. Social media data, particularly geotagged tweets, are valuable and cost-effective resources for near real-time spatial and spatiotemporal analyses. These data can help public health decision-makers analyze people’s movements or behaviors, especially during an outbreak, for prompt interventions. Nguyen et al. [63] employed geotagged Twitter data collected between April 2015 and March 2016 across the continental US. They randomly selected a sample of 1% of collected data, nearly 80 million tweets of over 603 million unique users, to predict health outcomes. They found that tweets around food and physical activities were associated with lower premature mortality and obesity. These data types are not as reliable as CDC or governmental published data, and are susceptible to bias. The accuracy and quality of collected data should be carefully examined before conducting any analysis or conclusion. As this review showed, these data can be helpful for COVID-19 vaccination research, particularly for examining people’s opinions regarding vaccination, such as vaccine hesitancy [12,13]. However, analysis of vaccination using Twitter data might be underrepresented, and is prone to misreporting, as people may just express their opinion towards COVID-19 vaccination instead of receiving a shot.

In a small number of spatial modeling of COVID-19 vaccination studies, the health outcome has been classified by potential confounders, such as socio-demographic status, age group, and gender. To reveal the true relationship between vaccination rate and the determinants, it is vital to account for potential confounders during study design via stratification, randomization, restriction, or matching [64]. However, before adjusting for confounding, the criteria for a potential confounder should be thoroughly investigated to avoid the introduction of extra bias due to an over-adjustment for variables that do not meet all confounding requirements [65].

There are two significant limitations in this study that should be acknowledged. Although two independent researchers carefully screened articles for selection and data extraction, they likely missed relevant and high-quality articles. Moreover, non-English articles were excluded which might have contained helpful information that could be incorporated in this review. Further spatial analysis of COVID-19 vaccination studies should carefully assess data quality, MAUP, and spatial dependence issues. Moreover, spatial and spatiotemporal analyses with robust foundations are suggested to provide a deeper understanding of COVID-19 vaccination research from spatial perspectives.

## 5. Conclusions

In summary, as COVID-19 vaccination data are becoming more available, the body of literature analyzing the spatial distribution of vaccination from a spatial perspective is also growing. However, our overview indicated that most spatial and spatiotemporal analyses utilized preliminary and non-robust techniques, predominantly based on vaccination surveillance. At the same time, little attention has been given to vaccination community profiling. Moreover, few studies addressed data quality, MAUP, and spatial dependence, which might bias their findings. Future sophisticated spatial analyses of COVID-19 from different parts of the world are necessary to minimize these drawbacks, and to provide a deeper spatial understanding of vaccination leading to more reliable information for public health decision-makers. The data used in each study refer to a relatively short period, which might not lead to a robust conclusion. Therefore, as vaccination data in the following months become more available, an updated review is recommended.

## Figures and Tables

**Table 1:** Articles’ search strategy in PubMed.

Theme	Keywords/MeSH Terms
Spatial Analysis	Keywords: “Geospatial” OR “Spatio-Temporal” OR “Geocode*” OR “Spatial Autocorrelation” OR “Georeference*” OR “spatial analysis” OR “Spatial inequality” OR “Spatial Dependency” OR “Space-Time” OR “Spatial Temporal” OR “Spatiotemporal” OR ” GIS” OR “spatial access*” OR “Geographic*” OR “Geographical mapping” OR” Geographic mapping” OR “Geographical information system*” OR “Geographic Information System*” OR “Spatio temporal” OR “space time” OR “Geographical distribution*” OR “Geographic distribution*” OR “spatial statistics” OR “Spatial hotspot*” OR “Spatial Cluster*” OR “Geographic cluster*” OR “Geographic hotspot*” MeSH terms: Geographic Information Systems OR Spatial analysis OR Geographic Mapping OR Spatio-Temporal Analysis
COVID-19	Keywords: “COVID-19” OR “Coronavirus” OR “nCoV Infection” OR “SARS-CoV-2” OR “COVID19” MeSH terms: COVID-19
Vaccination	Keywords: “Vaccine*” OR “Vaccination” MeSH terms: COVID-19 Vaccines

Note: * indicates any stacking character after the keyword was also considered as the keyword.

## Data Availability

All relevant data were included in the manuscript.

## Supplementary Materials

The following are available online at https://www.mdpi.com/article/10.3390/ijerph182212024/s1, File S1: Search strategies, File S2: Preferred Reporting Items for Systematic reviews and Meta-Analyses extension for Scoping Reviews (PRISMA-ScR) Checklist, File S3: A summary of key findings related to each category
