# Supplementary material for: Spatial Analysis of COVID-19 Vaccination: A Scoping Review
Source: Int J Environ Res Public Health. 2021 Nov 16;18(22):12024. doi: 10.3390/ijerph182212024 (PMC8624385; doi:10.3390/ijerph182212024)
Supplement: Supplementary file 1 [file ijerph-18-12024-s001.zip › Supplementary File S1.pdf]

Search Strategies:

1- PubMed Search Strategy: (94 articles on 21 September 2021)

((((COVID-19 Vaccines[MeSH Major Topic]) OR (vaccine*[Title/Abstract])) OR (Vaccination[Title/Abstract])) AND ((((("nCoV Infection"[Title]) OR (COVID-19[Title])) OR (Coronavirus[Title])) OR (SARS-CoV-2[Title])) OR (COVID19[Title])) OR (COVID-19[MeSH Major Topic]))) AND ((((((((((((((((((((((((((((("spatial access*"[Title/Abstract]) OR (Spatiotemporal[Title/Abstract])) OR ("Spatial Temporal"[Title/Abstract])) OR ("Spatio-Temporal"[Title/Abstract])) OR (Georeference*[Title/Abstract])) OR (Geocode*[Title/Abstract])) OR (Geographic*[Title])) OR ("Geographical information system*"[Title/Abstract])) OR ("Geographic Information System*"[Title/Abstract])) OR ("Spatio temporal"[Title/Abstract])) OR ("Geographical distribution*"[Title/Abstract])) OR ("Geographic distribution*"[Title/Abstract])) OR ("Geographic hotspot*"[Title/Abstract])) OR ("Geographic cluster*"[Title/Abstract])) OR ("Spatial Cluster*"[Title/Abstract])) OR ("Spatial hotspot*"[Title/Abstract])) OR ("spatial statistics"[Title/Abstract])) OR ("Geographic Information Systems"[MeSH Major Topic])) OR (Spatial analysis[MeSH Major Topic])) OR (Geographic Mapping[MeSH Major Topic])) OR (Spatio-Temporal Analysis[MeSH Major Topic])) OR ("Space time"[Title/Abstract])) OR ("Space-Time"[Title/Abstract])) OR ("spatial analysis"[Title/Abstract])) OR ("Spatial Dependency"[Title/Abstract])) OR ("Spatial inequality"[Title/Abstract])) OR ("Spatial Autocorrelation"[Title/Abstract])) OR (Geospatial[Title])) OR (GIS[Title])) OR ("Geographical mapping"[Title/Abstract])) OR ("Geographic mapping"[Title/Abstract]))

2- Scopus Search Strategy: (238 articles on 21 September 2021)

(TITLE-ABS-KEY ("Spatio-Temporal" OR geocode* OR "Spatial Autocorrelation" OR georeference* OR "spatial analysis" OR "Spatial inequality" OR "Spatial Dependency" OR "Space-Time" OR "Spatial Temporal" OR spatiotemporal OR "spatial access*" OR "Geographical mapping" OR "Geographic mapping" OR "Geographical information system*" OR "Geographic Information System*" OR "Spatio temporal" OR "space time" OR "Geographical distribution*" OR "Geographic distribution*" OR "spatial statistics" OR "Spatial hotspot*" OR "Spatial Cluster*" OR "Geographic cluster*" OR "Geographic hotspot*") OR TITLE (geospatial OR gis OR geographic*)) AND (TITLE (covid-19 OR coronavirus OR "nCov Infection" OR sars-cov-2 OR covid19)) AND (TITLE-ABS-KEY (vaccine* OR vaccination)) AND (LIMIT-TO (PUBYEAR , 2021) OR LIMIT-TO (PUBYEAR , 2020))

3- Web of Science Search Strategy: (64 articles on 21 September 2021)

(TI= (covid-19) OR TI= (coronavirus) OR TI= ("nCov Infection") OR TI= (sars-cov-

2) OR TI= (covid19)) AND (
TI= (vaccine*) OR TI= (vaccination) OR AB= (vaccine*) OR AB= (vaccination) OR AK=(vaccine*) OR AK=(vaccination)) AND (TI= ("Spatio-
Temporal") OR TI=(geocode*) OR TI=("Spatial Autocorrelation") OR TI=(georeference*) OR TI=("spatial analysis") OR TI=("Spatial inequality") OR TI=("Spatial Dependency") OR TI=("Space-
Time") OR TI= ("Spatial
Temporal") OR TI=(spatiotemporal) OR TI=("spatial access*") OR TI=("Geographical mapping") OR TI=("Geographic mapping") OR TI=("Geographical information system*") OR TI=("Geographic Information System*") OR TI=("Spatio temporal") OR TI=("space time") OR TI=("Geographical distribution*") OR TI=("Geographic distribution*") OR TI=("spatial statistics") OR TI=("Spatial hotspot*") OR TI=("Spatial Cluster*") OR TI=("Geographic cluster*") OR TI=("Geographic hotspot*") OR TI=(geospatial) OR TI=(gis) OR TI=(geographic*) OR AB= ("Spatio-
Temporal") OR AB=(geocode*) OR AB=("Spatial Autocorrelation") OR AB=(georeference*) OR AB=("spatial analysis") OR AB=("Spatial inequality") OR AB=("Spatial Dependency") OR AB=("Space-
Time") OR AB= ("Spatial
Temporal") OR AB=(spatiotemporal) OR AB=("spatial access*") OR AB=("Geographical mapping") OR AB=("Geographic mapping") OR AB=("Geographical information system*") OR AB=("Geographic Information System*") OR AB=("Spatio temporal") OR AB=("space time") OR AB=("Geographical distribution*") OR AB=("Geographic distribution*") OR AB=("spatial statistics") OR AB=("Spatial hotspot*") OR AB=("Spatial Cluster*") OR AB=("Geographic cluster*") OR AB=("Geographic hotspot*"))

Indexes=SCI-EXPANDED, SSCI, A&HCI, ESCI Timespan=2020-2021
