# Supplementary material for: Spatial Analysis of COVID-19 Vaccination: A Scoping Review
Source: Int J Environ Res Public Health. 2021 Nov 16;18(22):12024. doi: 10.3390/ijerph182212024 (PMC8624385; doi:10.3390/ijerph182212024)
Supplement: Supplementary file 1 [file ijerph-18-12024-s001.zip › Supplementary File S3.pdf]

Study group	Study sub-group	Number of articles (%)	Summary of approaches	Summary of results
Disease surveillance	Vaccine mapping	35 (97.22%)	The articles mainly represented geospatial distribution of vaccination, the output of predictive analyses or models. The employed maps were usually heat map, choropleth map, or inset map.	Public sentiment on COVID-19 vaccines varies significantly over time and space.<br>Twitter discourse regarding COVID-19 vaccines in the US varies significantly across different communities and changes over time.<br>The maps could identify geographic hotspots with low vaccination rates and persistent disease rates over time.
	Vaccine Modelling	16 (44.44%)	The articles in this category utilized geostatistical and modeling techniques such as agent-based models based on individual, socioeconomic and environmental variables.	Unlike the influenza virus or HIV viruses, SARS-CoV-2 has a low mutation rate, making developing an effective global vaccine very likely.<br>To reduce transmission, students should be prioritized for vaccination rather than retired older people and preschool-aged children.<br>During the planning and executing vaccine allocation, especially in the early stages of distribution, it is critical to evaluate which communities can benefit from the limited number of vaccines.

Risk Analysis	Risk Analysis	7 (19.44%)	The articles mainly analyzed the risk of vaccination (assessment, management, communication, or monitoring). Also, they examined the risk regarding demographic and socioeconomic factors including age, race, occupation, and income level. Moreover, distribution and allocation of vaccines for high-risk groups was analyzed.	Blacks and Hispanics have been disproportionately affected by COVID-19 in the US.<br>Receiving an influenza vaccine is of particular importance to mitigate the risk associated with overlapping influenza and COVID-19 infections.<br>National and subnational socioeconomic indicators and burden of disease estimates can potentially be leveraged to allocate vaccines optimally to reduce severe outcomes.<br>There are noticeable demographic and geographical disparities in vaccine acceptance across the US.
Health Access and Planning	Access to Vaccine Services	8 (22.22%)	The articles assessed the accessibility of different social groups to the healthcare facilities that provide vaccination.	Around 18% of Brazil's elderly population lives more than 4 kilometers from a vaccination point.<br>Even though inequalities appear at the metropolitan scale, mobile and walk-up sites improve both coverage and accessibility to COVID-19 testing services for hard-to-reach populations.<br>Several areas with significant travel times to potential vaccine delivery sites have an elevated risk of COVID-19 disease and severity.

				Spatial accessibility to vaccination services varies across Aotearoa and appear better in major cities than in rural regions.<br>Equitable COVID-19 vaccination services in metropolitan areas should also include transportation networks and spatial access in modeling.
	Vaccine Resource Management	16 (44.44%)	The articles provided vaccine allocation strategies and measured inequality in vaccine distribution,	Neighborhood-level geospatial data would continue to aid public health in striving toward a greater and more equitable health system.<br>Geographical information system technology allows practitioners to design efficient distribution scenarios rapidly.<br>Considering space in optimizing vaccines allocation is vital. Space & age strategy greatly improves the effectiveness of vaccine usability.
Community Health Profiling	Community Health Profiling	2 (5.55%)	In this category, the relationship of COVID-19 prevalence with other vaccination coverages such as Bacillus Calmette–Guérin (BCG) and influenza were assessed.	A high prevalence of COVID-19 is identified in the geographic areas with low coverage of BCG vaccination.<br>There are significant positive correlations between invasive pneumococcal disease and COVID-19 rates, while significant negative correlations are found between pneumococcal vaccination and COVID-19 rates.
